# Whispers of Resilience: A phenomenological exploration of maternal journeys with children affected by Downs Syndrome in Quetta, Baluchistan

**DOI:** 10.12669/pjms.41.3.9710

**Published:** 2025-03

**Authors:** Ambica Devi, Lubna Ansari Baig, Mehjabeen Musharaf, Hira Tariq

**Affiliations:** 1 Dr. Ambica Devi, MSPH. World Health Organization, Quetta, Pakistan; 2 Prof. Lubna Ansari Baig, FCPS, PhD. University of Lahore, Karachi, Pakistan; 3 Mehjabeen Musharaf, MSPH. Westman Immigrant Services, Brandon, Canada; 4 Dr. Hira Tariq, MSPH Aga Khan University Hospital, Karachi, Pakistan

**Keywords:** Downs Syndrome, Mother’s journey, Phenomenological study, Quetta Baluchistan

## Abstract

**Background & Objective:**

Down’s syndrome, a chromosomal disorder, with an incidence of approximately one in 800 live births globally affects physical and intellectual development of a child, having greatest impact on mothers. This study explored the challenges faced by the mothers of children with Down’s Syndrome related to their upbringing at different stages of life.

**Methods:**

Qualitative research was conducted using Phenomenological approach from May 2021- April 2022 in Quetta, Baluchistan. Mothers were selected through purposive sampling from institutional records of Wilderness School, Quetta, the only private school for special children. Five In depth Interviews and two Focus Group Discussions were conducted with 17 mothers using a semi structured interview guide. Data collected in audio files was transcribed verbatim into English language. Analysis was done by two researchers independently.

**Results:**

The themes included, Experience of Giving birth to a child with Down’s Syndrome, Social support system, Personal Challenges, Experiencing Worrisome thoughts about child’s future and Experiencing personality changes. The findings imply that giving birth and bringing up a child with DS is a life-changing process. Social stigmatization, lack of support by family, in-laws, relatives and financial constraints to afford schooling and rehabilitation make it challenging for mothers to provide adequate attention and developmental opportunities to the child. Mothers compromise on their health, career, social life, social status and relationship with other family members.

**Conclusion:**

Raising children with Down syndrome is a life changing process and the absence of prenatal diagnosis in regions like Quetta and limited counseling from concerned doctors leaves mothers unprepared. This results in care givers reliance on internet resources for developing understanding about the syndrome and child care.

## INTRODUCTION

Down’s syndrome (DS) is a common chromosomal disorder, which occurs because of presence of an additional copy of chromosome 21 and is also called trisomy 21. DS as compared to other chromosomal disorders is known as the most common genetic cause of intellectual disability (ID) with an incidence of approximately 1/800 live births worldwide.^1^ It affects both physical and intellectual development and produces characteristic facial features such as flattened face and nose, narrow upward slanting eyes, small head, ears and mouth.^2^ DS is also associated with conditions like Alzheimer’s disease, acute childhood leukemia, congenital heart malformations, and immunologic abnormalities.^1^,^3^,^4^

Children with DS usually have considerable cognitive impairments, the average intelligence quotient (IQ) of children with DS is almost 50, but levels ranging between 30 to 70 are noted, which is almost equal to mental ability of 8-9 years old child.^5^ Cognitive domains that are affected in individuals with DS include speech abilities, delayed learning, deficit memory, executive function, and motor coordination that shape the intellectual disability of the syndrome.^1^,^6^

The challenges of caregiving for children with Down syndrome are substantial, as they stem from developmental delays and limitations in daily self-care activities such as dressing, personal hygiene, and additional needs related to health, education, and leisure.^7^ These demands often fall disproportionately on the primary caregiver, who bears most of the responsibility without receiving any financial compensation. In most families, this role is taken on by mothers, making them particularly vulnerable to the burden of caregiving.^7^ A study in Sergipe found mothers as primary caregivers for children with and without DS faced significant burdens (p < 0.001). Those with DS were more dependent on caregiver support.^7^ They bear all the physical, emotional, and financial burden and minimize their social and leisure activities.^7^ A cross-sectional study in Nepal involving 96 mothers of children with DS found that a significant majority (77%) experienced a high level of caregiving burden, and nearly 90% (89.6%) felt overwhelmed by their child’s condition.^8^

Mostly quantitative studies have determined the experiences of families having a child with intellectual disability^9^ while very few have used a qualitative research approach^10^ to highlight the experiences of parents with DS child. As most of the research is done in affluent western countries so this research helped us to develop understanding related to the experiences of mothers having a child with DS within a Pakistani family context, a low- and middle-income country. The phenomenological approach helps to explain that the same event may have several different aspects giving a detailed meaning to the phenomena. Through this study we explored the challenges faced by the mothers of children with DS in their upbringing at different stages of life.

## METHODS

Qualitative research using a phenomenological approach was conducted in Quetta, Baluchistan from May 2021 to April 2022. Through this approach we focused on the unique experiences of mothers and the various influences that shape these experiences. Quetta is the capital and largest city of Baluchistan with a population of three million. Half of this population lives in informal settlements known as “Kachi Abadis”. The area is prone to natural disasters such as earthquakes, landslides, and floods. Additionally, the city faces terrorism, civil unrest, and issues with refugees. These problems, along with a weak healthcare system, make Quetta’s population vulnerable, particularly affecting the availability of health services for mothers and children.^11^

### Ethical Approval:

The study was approved by the institutional review board (JSMU/IRB/2021/-409; dated May 5, 2021) and written informed consent was taken from all the participants.

The mothers of children with DS were identified from institutional records of Wilderness School, Quetta. The age of the participants ranged from 20 – 55 years with the mean age of 35 years. IDIs were conducted at the residence of mothers after seeking their permission and FGDs were conducted at Wilderness school. Purposive sampling was employed, involving two FGDs with six mothers each and five IDIs. Interviews continued until data saturation was reached, indicated by consistent responses and theoretical data exhaustion. In total, 17 mothers participated in the study. The Wilderness school is the only private school for DS children in Quetta. Government school was also approached but no positive response was given and permission was denied. Mothers having any physical or mental disability or not providing written informed consent were excluded from the study. A semi-structured interview guide was used to collect data on the experiences of mothers. All the collected data in audio files was transcribed verbatim into English language. Data was analyzed by two researchers separately consisting of Principal investigator and the Co-investigator. Emerging themes were identified and carefully documented with consensus of both the investigators.

## RESULTS

**Fig.1:**
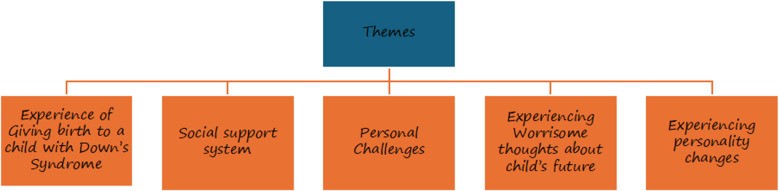
Themes identified from the transcripts (5 IDIs and 2 FGDS)

Six themes were generated after the analysis which included, Experience of Giving birth to a child with DS, Social support system, Personal Challenges, Experiencing Worrisome thoughts about child’s future and Experiencing personality changes (Fig.1).

### 1. Experience of Giving birth to a child with Down’s syndrome:

The study participants shared that they were unaware about the possibility of giving birth to a child with any congenital problem. This included mothers who regularly followed all antenatal visits at health facilities. All mothers reported psychological trauma and mental stress that they experienced when they learnt about the diagnosis of the child. Social stigmatization was uniformly mentioned by all mothers. Most women experienced disheartening, negative and harsh responses about giving birth to a child having DS from family members, friends and in-laws. A few also experienced positive responses from their friends and family. Some mothers reported strengthening in their faith while accepting the condition of the child as God`s Will.

*“**I used to have regular checkups and ultrasound during my pregnancy but I was never told that my child is not normal”. (39 years old, mother of four children)***

***“My family was not supportive especially my in-laws` reaction was not good. They said that I am reason for having the child with Down`s syndrome as there was no child before my son in their whole family who had Down`s syndrome.” (33 years, mother of 4 children)***

***“My friends and relatives never made me feel that I have special child, they all love him a lot and give equal importance to him as they give to my other children.” (34 years, mother of 3 children)***

***“It is very difficult for a mother to face everyone’s questioning eyes as your child is different from other normal children.” (29 years, Mother of 2 children***)

*“All my family including my husband and in-laws were really sad when the reality was revealed but we accepted it as Will of God.” (55 years, Mother of 3 children)*

*“I am lucky that God has chosen me to take care of a child who is a special gift of God.” (27 years, Mother of 3 children)*

### 2. Experiences with existing social support system:

Most of the mothers experienced difficulties in upbringing of the child with DS because of unavailability of formal and informal services to fulfill the special needs of children. None of the mothers participating in this study denied negative social attitudes and strange responses they experienced from people in their social circle. Moreover, they reported a lack of healthcare services, education services and social support programs for children with special needs.

*“There are no facilities in Quetta; no physiotherapists, no speech therapists so I really got depressed and started to worry about her health issues.” (38 years, Mother of 5 children)*

*“Other challenge I faced was to find out a school where I can admit my child as I visited Government school for special children. The school was in miserable condition and I was not satisfied at all from its atmosphere and standard of education.” (34 years, Mother of 3 children)*

*“People show sympathy when I go into different social gatherings. Some also stare at my child and make me feel discriminated as she is not normal.” (27 years, Mother of 3 children)*

### 3. Personal Challenges:

In our culture giving birth and bringing up a child with DS affects the social life of mother, as she is supposed to be the primary caregiver for the child. Hence, a mother might experience lack of socialization or even complete social isolation. The mothers who were initially working with family support ended up quitting their jobs. Similarly, women who were entrepreneurs lost their clients and business because of taking care of the child during work hours. Most of the study participants shared that they experienced financial challenges while upbringing of their child with DS.

*“I cannot leave her alone at home and if I have to go somewhere then either I have to take her with me or make sure to leave her with someone with whom she is comfortable. Therefore, I can’t socialize the way I used to be before her birth.” (33 years, Mother of 4 children)*

*“Other major challenge was to keep a balance between my family and my job as most of my attention was towards my son so I had to quit job.” (34 years, Mother of 3 children)*

*“My business suffered a lot as I couldn’t give time to my clients as they get distracted if he is with me.” (37 years, Mother of three children)*

*“Due to her multiple surgeries we had to borrow money. My father also supported me financially as we faced huge financial crisis.” (38 years, Mother of 5 children)*

### 4. Experiencing Worrisome thoughts about child’s future:

Experiencing worrisome thoughts about child`s health, development, education, career, family life and support system if mother is no more, emerged as a uniform experience by all the mothers.

*“I am training her in a way that she at least takes care of herself and not become a burden on others.” (35 years, Mother of 6 children)*

*“I also wish to send her to Special Olympics as she is very flexible but I want someone to guide me about Special Olympics.” (32 years, Mothers of 4 children)*

*“I am helpless and extremely worried about his future.” (29 years, mothers of 2 children)*

*“I think a lot about her future and get really worried that if something happens to me then who will take care of her. There is a lot of difference in upbringing of mother and father. No one else can take care of her as much as I do,” (33 years, Mother of 4 children)*

### 5. Experiencing personality changes:

Mothers shared different experiences of change in tolerance level, some reporting increase in their tolerance whereas some experienced decrease in their tolerance as their maximum attention and energy is diverted towards their child with special needs which is tiring and draining for them.

*“I have become very sensitive and I am not able to take stress any more. If even someone shouts on him, I become upset and get disturbed.” (36 years, Mother of 5 children)*

*“I have become more confident and my patience and tolerance level has increased. My belief in Allah has become stronger”. (36 years, Mother of 5 children)*

## DISCUSSION

A very surprising and major finding of this study was that none of the mother was ever told about the risk of delivering a child with Down`s syndrome. These findings are in concordance with the study conducted in China and results of the study revealed that birth of their child having down’s syndrome was a surprise for them as none of them were provided timely information regarding the syndrome by health care providers.^12^ Other studies found similar patterns of diagnosis of Down`s syndrome as none of the child was diagnosed in the antenatal period while only few were diagnosed to have the syndrome at the time of birth. The late diagnosis was also recognized to increase the stress among the parents of the child with Down’s syndrome.^13^,^14^ This aligns with the current situation of Pakistan, which being a low- and middle-income country (LMIC), faces significant disparities in healthcare provision.^15^

Our investigation revealed that many women faced social stigmatization, unfairly held responsible for having a child who didn’t fit societal norms. This resonates with a broader study highlighting how stigma and intolerant behaviors can engender negative emotions and feelings of worthlessness in mothers.^16^ Some women were unjustly labeled as “cursed by God” or blamed for their child’s condition—this finding resonates with a study in which mothers who gave birth to a child with down’s syndrome were blamed for such a birth to be either a curse by God or ancestors who may not be happy with the personal conduct of the mother.^17^

Furthermore, a common challenge faced by all these mothers was the scarcity of affordable, quality education and healthcare institutions providing education and development opportunities for their child. Similar findings were reported from Zambia and Kuwait, where accessing quality education and social participation and lack of healthcare facilities posed significant challenges.^18^,^19^

The birth of a child with DS had a substantial negative impact on the mothers’ quality of life, imposing physical, mental, and financial burdens. Almost every mother experienced severe stress and anxiety and many of them developed depression. This finding is in line with previous evidence reporting shock and depression as a natural response followed by acceptance for the situation.^20^

Many mothers left their jobs to care for their child, while others sought employment to better support their child’s needs and education. These findings are consistent with previous studies highlighting similar experiences among caregivers of children with DS.^21^-^23^

Interestingly, the mothers in our study found that caring for a child with DS demanded extraordinary strength and effort. This is consistent with findings of studies reporting more tolerant and resilient in the face of adversity.^22^,^24^,^25^

### Recommendations:

Based on the study findings we have devised some recommendations which include Provision of quality antenatal care must be ensured with availability of perinatal screening for congenital anomalies by strengthening of the healthcare system and training of healthcare providers in early detection and diagnosis of DS especially in Government hospitals. Timely counselling of the parents, especially expectant mothers shall be done by health care providers. Public awareness campaigns and community programs can play a vital role in changing negative perceptions and attitudes of society. Governments and educational institutions must prioritize the development of accessible and affordable special education programs. These schools/ institutions should be regularly monitored for providing quality education and training to students with special needs. Further research should be done for identifying better quality of care for children with downs syndrome and other disabilities.

### Strengths of study:

This study offers a deep insight into a marginalized population including mothers from all different socioeconomic strata. Findings of this study can have strong practical implications by highlighting the dire need of tailored behavior change, interventions to improve the socio-cultural acceptability for children with disabilities as well as to ensure provision of support to the mothers at all levels.

### Limitations:

It include single center study and the selection of participants were also from one institute.

## CONCLUSION

Our study highlights the life-altering journey of mothers raising children with Down syndrome, extending its impact to entire families. The absence of prenatal diagnosis in regions like Quetta and limited counseling leaves mothers unprepared, relying on internet resources. Social stigmatization, family unsupportiveness, financial constraints, and lack of access to education compound the difficulties, fostering isolation. Mothers grapple with the uncertainty of their child’s future, compromising their physical and mental health, careers, and social lives.

### Authors’ contributions:

**AD:** Conception, design, data collection.

**LAB:** Conception, design, review.

**MM:** Data analysis and review.

**HT:** Data analysis, manuscript write-up and responsible for the accuracy of the study.

All authors have read and approved the final version.
